# From Micronutrients to Potentially Toxic Elements: Physiological Responses of *Canavalia ensiformis* to Copper and Iron

**DOI:** 10.3390/metabo15110706

**Published:** 2025-10-29

**Authors:** Nayane Cristina Pires Bomfim, Tassia Caroline Ferreira, Jailson Vieira Aguilar, Maiara Luzia Grigoli Olivio, Beatriz Silvério dos Santos, Isabella Fiorini Carvalho, Aline Renee Coscione, Lucas Anjos Souza, Liliane Santos Camargos

**Affiliations:** 1 Plant Metabolism Physiology Laboratory, Department of Biology and Zootechny, School of Engineering, São Paulo State University (UNESP), Ilha Solteira 15385-086, SP, Brazil; nayanecristinapires@gmail.com (N.C.P.B.); aguilarsbio@gmail.com (J.V.A.);; 2 Instituto Agronômico de Campinas, Campinas, São Paulo 13020-902, SP, Brazil; aline.coscione@sp.gov.br; 3 Instituto Federal Goiano, Campus Rio Verde, Rio Verde 75901-970, GO, Brazil

**Keywords:** tolerance, biomass, metal ion toxicity, translocation, accumulation, multivariate analysis

## Abstract

**Background**: The increase in potentially toxic elements (PTEs) in the soil is worrying, especially in agricultural soils due to the bioaccumulation factor. Copper (Cu) and iron (Fe) are micronutrients, responsible for important functions in the plant body, but the high availability of these elements in the soil can cause soil contamination and toxicity in plants; consequently, they can be considered PTEs. **Objectives**: The focus of this study is to understand the physiological responses (pigments, gas exchange, growth, biomass, accumulation) of *Canavalia ensiformis* to high levels of Cu and Fe in the soil, in isolation, and to identify which PTE is most harmful to its development. **Methods**: Two experiments (Cu and Fe) were conducted simultaneously in a greenhouse. Treatments of 50, 150, 250, and 350 mg dm^−3^ of soil for each element (CuSO_4_*5H_2_O and FeSO_4_*7H_2_O) were incorporated into the soil (Oxisol) of each experimental unit (4 dm^3^ pot), in addition to the control. *C. ensiformis* seeds were sown directly in soil enriched with Cu and Fe, respectively, and after emergence they were cultivated for 90 days. **Results**: Changes in chlorophyll levels caused direct effects on gas exchange, shoot biomass, root development, nodulation, and total plant biomass. The tolerance of the species is dependent on chlorophyll levels and gas exchange. There was accumulation of both PTEs in the roots and low translocation to the shoot. **Conclusions**: The plants were tolerant to Fe treatments; however, they were not tolerant to Cu treatments (T150–T350). Excess Cu was more detrimental to plant development.

## 1. Introduction

Plants require essential elements throughout their cycle to develop, from germination to reproduction. Some elements are required in low concentrations, called micronutrients or essential trace elements, such as manganese (Mn), iron (Fe), copper (Cu), zinc (Zn), and boron (B), among others. Cu and Fe are commonly referred to as heavy metals due to their density exceeding 5.0 g cm^−3^. However, the International Union of Pure and Applied Chemistry (IUPAC) [1] recommends using the term “trace elements (TEs)”, rather than “heavy metals”, emphasizing their biological relevance at low concentrations. These trace elements occur in natural and/or disturbed environments [2].

In uncontaminated environments, TEs typically occur in low concentrations in soils, generally below 1000 mg kg^−1^ or <100 ppm [3,4], posing minimal toxicity risks. However, for certain TEs, the margin between essential and toxic levels for plants can be narrow, fluctuating within a range of a few milligrams per kilogram (mg kg^−1^) [2]. This depends on the specificity of the element, the physicochemical conditions of the soil (increased soil acidity, organic matter, and clay content), and/or the plant species [5]. The distinction between TEs and potentially toxic elements (PTEs) is primarily based on their concentration in the environment or within plant tissues [5,6]. While TEs are essential for plant growth and physiological function, they become PTEs when their concentrations exceed critical thresholds, leading to phytotoxic effects. This transition depends on the element’s bioavailability, soil physicochemical properties, and plant species [2].

Cu and Fe participate and share important functions within the body of plants, due to their ease in accepting and donating electrons (redox properties), necessary for the catalysis of biological reactions such as photosynthesis, respiration, and elimination of reactive oxygen species (ROS) [7,8,9,10]. At ideal concentrations, Cu and Fe are essential for normal development in almost all organisms, but at high concentrations these TEs can be considered PTEs [5]. The high availability of Cu and Fe in the culture medium or accumulated in high concentrations in the plant body (cells) or in certain organelles can be toxic and cause damage to plants, such as reduced biomass and productivity [11,12].

The significance of studying Cu and Fe lies in their dual nature: essential for metabolic function yet harmful when in excess. Copper (Cu) concentrations in mature leaf tissues are considered sufficient between 5 and 30 mg kg^−1^, tolerable in agronomic crops within the range of 5–20 mg kg^−1^, and potentially toxic when reaching levels from 20 to 100 mg kg^−1^ according to Kabata-Pendias [4]. In soils, Cu values above 25–40 mg kg^−1^, especially in acidic conditions (pH < 5.5), can induce toxicity, with permissible limits suggested at 20 mg kg^−1^ for soil and 30 mg kg^−1^ for food crops [13,14,15,16]. Iron (Fe), on the other hand, typically ranges from 30 to 550 μg L^−1^ in soil solution but may exceed 2000 μg L^−1^ in highly acidic soils [16]. Natural Fe concentrations vary across plant types, from 30 to 100 mg kg^−1^ in cereal grains, 18 to 1000 mg kg^−1^ in forage plants, and <1 to 70 mg kg^−1^ in foods [16]. Other authors report that concentrations above 500 mg kg^−1^ of dry mass in leaf tissues cause phytotoxicity [17,18]. High concentrations of Fe can be toxic, depending on the species, but these levels are rarely mentioned in plants and in different types of soil.

*Canavalia ensiformis* (L.) DC is an annual, fast-growing, rustic herbaceous legume from the Fabaceae family, native to Central America and widely distributed in tropical and subtropical regions of South America, Asia, the South Pacific, and West Africa, among other areas [19]. This wide distribution is related to its remarkable adaptability to different soil types, even with low fertility, as well as its resistance to adverse environmental conditions such as high temperatures, drought, salinity, acidity, herbivory, and metal stress [20,21,22,23]. The species has high biomass production (4 to 8 t ha^−1^ of dry matter in 120 days) [24,25], performs biological nitrogen fixation (BNF) in symbiosis with rhizobia, and may associate with mycorrhizal fungi [26]. Due to its physiological robustness, growth capacity in degraded environments, and tolerance to abiotic stress, *C. ensiformis* stands out as a promising model for studies on tolerance and phytoremediation against trace elements such as copper (Cu) and iron (Fe), which act simultaneously as micronutrients and potentially toxic elements (PTEs).

Legumes comprise a group of very important species for food. They have been the target of PTE remediation studies to improve soil fertility and promote soil decontamination and revegetation [27,28]. Several studies have investigated the potential of *Canavalia ensiformis* for phytoremediation of contaminants such as sulfentrazone [29], hydrocarbons [30], zinc [31], and combinations of multiple elements in soil [32,33], often with the help of assisted technologies such as inoculation with mycorrhizae, rhizobia, or organic fertilizers [33,34,35,36,37]. Although these approaches increase the species’ ability to remediate contaminated soils, few studies have evaluated its specific physiological responses to copper (Cu) and iron (Fe) in isolation and without external stimuli. This study fills this gap by comparing the effects of these potentially toxic elements on photosynthetic performance, tolerance, biomass accumulation, and the uptake and accumulation of Cu and Fe, revealing the species’ intrinsic potential for phytoremediation under unassisted conditions, that is, without the addition of growth-stimulating factors.

Metal ion stress can present apparent signs of phytotoxicity, since these elements are associated with metabolic and ultrastructural changes in plants, such as respiration, photosynthesis, and stomatal opening [38]. These and other responses depend on the tolerance of each species to each PTE, and in addition, they depend on the available concentration of PTEs in the soil and the physicochemical properties of that soil. It is important to study each case (species) to identify the tolerance and impact of each PTE on the soil–plant dynamics.

Thus, this study aimed to evaluate, comparatively, the physiological parameters of *Canavalia ensiformis* in response to copper and iron by (a) evaluating the growth, tolerance, and photosynthetic responses of *Canavalia ensiformis* to concentrations of Cu and Fe in the soil; (b) identifying possible toxicity effects of both PTEs on the development of *Canavalia ensiformis*; (c) assessing which element is most detrimental to the development of *Canavalia ensiformis* regarding Cu and Fe; (d) assessing whether there is absorption, translocation, and accumulation of both elements in *Canavalia ensiformis.*

This study contributes to filling in research gaps in terms of the physiology of metal stress and the range of tolerated and toxic concentrations of *Canavalia ensiformis* to copper and iron (isolated effect), evaluating its physiological response without the use of assisted technologies. Furthermore, this work may inspire new experimental approaches, such as the effect of this combination of Cu and Fe elements; the evaluation of enzymatic and non-enzymatic antioxidant mechanisms; the use of assisted technologies to enhance phytoremediation; and field studies with the species. Because it is a species widely cultivated in tropical and subtropical regions, it has potential for further study and international applicability in phytoremediation strategies.

## 2. Materials and Methods

### 2.1. Soil Characterization

The soil used in this study, a typical Dystrophic Red Latosol according to the Brazilian Classification System [39,40] or Typic Haplustox (Oxisol) according to the classification of USDA Soil Survey Staff [41], was collected (0–40 cm deep layer) in the experimental area of the teaching, research, and extension farm (FEPE), animal production sector, Selvíria-MS (20° 20′ 24.9” S 51° 24′ 19.7” W). Two soil samples, consisting of 10 subsamples each, were removed for chemical characterization [42], soil density assessment using the volumetric cylinder method, and textural classification [43] according to Table 1. The soil was used under the conditions presented in Table 1; acidity correction was not carried out, fertilizers (organic or inorganic), rhizobia, and mycorrhizae were not added to the soil.

### 2.2. Copper and Iron Treatments

The trace elements evaluated in this study were Cu and Fe, separately. The Cu and Fe concentrations (treatments) tested were as follows: 0 (control), 50, 150, 250, and 350 mg dm^−3^ of soil, named T0, T50, T150, T250, and T350, respectively. These treatments and their respective concentrations were proposed based on the concentrations established by CONAMA Resolution nº 420/2009 [44], Kabata-Pendias [16], Batista et al. [45], and Bomfim et al. [46,47]. For more information, see Supplementary Material S1.

Stock solutions of copper sulfate pentahydrate (CuSO_4_*5H_2_O) and ferrous sulfate heptahydrate (FeSO_4_*7H_2_O) were prepared, separately. Volumes of 0, 20, 60, 100, and 140 mL of each stock solution were diluted with distilled water until the final volume of 400 mL was reached. A single application of either the copper or iron solution described (400 mL) was added to each experimental unit of 04 dm^3^ of soil for artificial contamination of the soil by copper (60 units) and iron (60 units), and then the soil was mechanically homogenized in a concrete mixer. Subsequently, samples were placed in transparent plastic bags, identified by the number of treatments and replications, corresponding to the experimental units, and incubated for 30 days, in a greenhouse, to stabilize the TE in the soil.

After incubation, composite soil samples were removed and subjected to chemical analysis for pH (4.8 ± 0.06) and plant available contents of Cu or Fe [48,49]. To determine copper and iron in the soil (available to plants), a 5 g soil sample was subjected to double-acid extraction solution (25 mL) of Melich 1 (HCl 0.05 M + H_2_SO_4_ 0.0125 M), in a soil/extractor 1:5 ratio, and the sample was stirred at a speed of 40 rpm in a horizontal circular shaker for five minutes [48,49].

The supernatant solution was separated from soil and submitted to ICP-OES. To determine semi-total micronutrient contents in soil samples after the treatments with the described solutions, microwave-assisted acid digestion (US-EPA3051 method) and inductively coupled plasma optical emission spectrometry (ICP-OES) were used [50]. The Cu and Fe quantification limits for either total or plant available content were estimated at 1.5 and 18 mg kg^−1^, respectively. The brand and model of the ICP-OES (FAAS, Agilent, Model 55, Santa Clara, CA, USA) and the microwave system were as follows: Varian, model ES710A, and CEM Corporation, model MARS 5 (CEM Corporation, Matthews, NC, USA), respectively. Inductively coupled plasma optical emission spectroscopy (ICP-OES) is a technique that measures the elemental composition of a sample by analyzing the light emitted by its atoms and ions, commonly used in agriculture for soil and sample analyses, especially nutritional multielement analysis.

The values for the bioavailable content or semi-total content of Cu and Fe in the soil were called TECsoil (mg kg^−1^) and were used to calculate the concentration (mg) of Cu and Fe available in 4 dm^3^ of after enrichment (treatments) with Cu and Fe in the soil (TEAC) (Equation (1)).

(1) $$TEAC=\left(TECsoil\times Vsoil\right)\times Dsoil$$

where Vsoil is the volume of soil used (dm^3^) and Dsoil is the soil density (kg dm^−3^).

### 2.3. Experimental Design

Two independent experiments, one for Cu and one for Fe, were conducted simultaneously in a greenhouse for 96 days from sowing to plant collection. For each element, five concentrations/treatments (treatment factors) were tested; each treatment was composed of six replications, consisting of one individual in each experimental unit, totaling 30 pots (4 dm^3^ of soil) or experimental units in a completely randomized design (CRD) per element.

The experiment was carried out in a greenhouse, under natural light and temperature conditions, with automatic sprinkler irrigation, in the experimental area of the Plant Metabolism Physiology Laboratory of the State University of São Paulo (Unesp), College of Engineering, Ilha Solteira-SP (20° 25′ 06.0″ S and 51° 20′ 29.7″ W), Brazil, between March and June 2021. The temperature and relative humidity conditions were an average temperature of 24.80 °C; a maximum temperature of 32.40 °C; a minimum temperature of 18.50 °C; and an average relative humidity of 69.20%. To clarify further, to compare the effects of copper and iron, two experiments were carried out, a CRD of 30 units for Cu and a CRD of 30 units for Fe, totaling 60 experimental units conducted simultaneously under the same conditions regarding water, light, and temperature in the greenhouse.

### 2.4. Choice of Plant Material

Seeds of *Canavalia ensiformis*, common cultivar, purity 99.3% and germination 91%, batch 01/2017, obtained commercially by BRSEEDS^®^ (BRSEEDS, Araçatuba, SP, Brazil), were used. The seeds were selected according to their size and shape, and no treatment was conducted on the seeds, such as phytosanitary treatment or the inoculation of rhizobia or mycorrhizal fungi [51]. Three seeds were sown in each experimental unit at a depth of 2.00 cm. After seedling emergence, they were thinned, and the experiment was conducted with one plant per pot for 96 days.

### 2.5. Gas Exchange Analysis

To determine the plants’ photosynthetic response, gas exchange was measured in each replication at 85 days after germination (reproductive stage), using a portable gas exchange analyzer, CIRAS-3 (Portable Photosynthesis System—PP Systems, Amesbury, MA, USA), according to the environmental conditions. Measurements were carried out on fully developed and apparently healthy leaflets in the 2nd or 3rd trefoil region below the apical meristem between 09 a.m. and 12:00 p.m., with an average daily temperature in the greenhouse of 22.05 °C for all treatments.

The following settings were applied for gas exchange: one leaflet inside the PLC3 universal leaf cuvette (window 18 × 25 mm), 390 µmol mol^−1^ reference CO_2_, 60% reference water (humidity), cuvette flow at 300 mL min^−1^, analyzer flow at 100 mL min^−1^, ambient light source, infrared radiation sensor for measuring leaf temperature (36.00 °C), with an average external radiation of 1238.73 µmol m^−2^ s^−1^, a leaf area of 4.50 cm^2^, a boundary layer resistance of 0.40 m^2^ s mol^−1^, stomatal proportion of 50%, with auto-zero system calibration [52,53].

The following parameters were analyzed and calculated based on the equations of Parkinson et al. [54]; Nijs et al. [55]; Cannel and Thornley [56]; and Zhang et al. [53]: photosynthetic rate (A, µmol CO_2_ m^−2^ s^−1^); transpiration (E, mmol H_2_O m^−2^ s^−1^); stomatal conductance (gs, mmol H_2_O m^−2^ s^−1^); water use efficiency (WUE, mmol CO_2_ mol^−1^ H_2_O); intrinsic water use efficiency (EIUA, µmol CO_2_ mmol^−1^ H_2_O); internal concentration of CO_2_ (Ci, µmol mol^−1^); ratio between internal and external carbon (CiCa: Ci/Ca, mol m^−2^ s^−1^); and carbon use efficiency (CUE: A/Ci, mol m^−2^ s^−1^).

The photosynthetic rate (A) is the rate at which CO_2_ is fixed into sugars by photosynthesis, which indicates the plant’s growth potential and productivity. The transpiration rate (E) is the loss of water from leaves via stomata, which is related to cooling and nutrient transport. Stomatal conductance (gs) is the degree of stomatal opening for gas exchange, which affects CO_2_ entry and water vapor exit. Water use efficiency (WUE) is the ratio of photosynthesis to transpiration, which measures the efficient use of water for carbon fixation. Intrinsic water use efficiency (EIUA) is the ratio of photosynthesis to stomatal conductance, which assesses physiological efficiency independent of water availability. Internal carbon concentration (Ci) is the level of CO_2_ within the leaf available for photosynthesis, which indicates the limitation or efficiency of carbon fixation. The internal-to-external-CO_2_ ratio (CiCa) is the comparison between internal (Ci) and atmospheric (Ca) CO_2_, which assesses the efficiency of CO_2_ capture and use. Carbon use efficiency (CUE) is the capacity to convert fixed carbon into biomass, which reflects growth and dry matter accumulation.

### 2.6. Collection and Determination of Photosynthetic Pigments

At the end of the 96 days of cultivation, the plant material was collected, separated into shoots (leaves and stem) and roots, washed in distilled water, and dried with paper towels. The stem length (SL, cm) was measured with a tape measure; the numbers of fully developed leaves (NL) and nodules (NN) were counted. The leaf area (LA, cm^2^) of the central leaflet was measured with a portable meter (Portable Leaf Area Meter, LI-3000C, COR Biosciences, Lincoln, NE, USA). The fresh weights (g) of the shoots (SFW), roots (RFW), legumes (LFW), and nodules (NFW) were determined. All plant growth measurements were performed according to Benincasa [57]. The root volume (RL, cm^3^) was measured by adding the root to a graduated cylinder with distilled water of a known value; from the displacement of the water and the equivalence of units (1 mL = 1 cm^3^), the root volume was obtained [58].

Fresh leaves were used to quantify chlorophylls (Chla, Chlb, and TChl) and total carotenoids (CAR) (mg g^−1^ fresh weight). Dimethyl sulfoxide (DMSO) was used to extract chlorophyll and carotenoids, and readings were made at 645 and 663 nm for chlorophylls and at 480 nm for carotenoids, according to Hiscox and Israelstam [59] and Wellburn [60], respectively.

### 2.7. Biomass and Estimation of Cu and Fe Content in the Plant

The remaining leaves, stems, and roots were packed in paper bags and dried in an oven with forced circulation at 60 °C for 72 h [61]. The dry weight (biomass, g) of leaves (LDW), stems (StDW), roots (RDW), and total biomass (sum of organs, TDW) was obtained, and then this material was ground in a Wiley mill (10-mesh sieve), adequately separated, and identified regarding replication, treatment, and organ.

The determination of Cu and Fe in plants was performed by nitro perchloric digestion (HNO_3_:HClO_4_, 2:1) by atomic absorption spectrophotometry: 250 mg ground and dried sample (root, stem, and leaf separately) with 3 mL of nitric acid + perchloric acid solution in a digestion block, where the temperature was gradually increased from 80 to 160 and then 210 °C. The resulting extract was adjusted to 25 mL with deionized water. Subsequently, readings were performed by atomic absorption AAS, make and model: Agilent SpectrAA-55B Atomic Absorption (Mulgrave, Victoria, Australia) [49,50]. The results for available concentration (mg Kg^−1^) of PTEs in the organs (TECleaves/stem/root) of the plants were used for phytoremediation calculations.

Calculations were performed to determine the phytoremediation potential: tolerance index (TI) [62]; transfer factor (TF) [4]; TE accumulation in plant organs [46,47]; and TE accumulation percentage in the root system (RAP%) and shoot (SAP%), based on the calculation of the TE translocation index [4].

The content data (TEC) of each element and the biomass of each organ (RDW, LDW, and StDW) were used to calculate the TE accumulation in roots (RAC), leaves (LAC), stems (StAC), and the entire plant (TAC).

The equations of TE accumulation in roots (RAC—Equation (2)), leaves (LAC—Equation (3)), stems (StAC—Equation (4)), and the whole plant or total plant (TAC—Equation (5)); tolerance index (TI—Equation (6)); transfer factor (TF—Equation (7)); TE accumulation percentage in the shoot (SAP%—Equation (8)); and root system (RAP%—Equation (9)) are listed below:

(2) $$RAC=\left(RDW\times TECroots\right)/1000;$$

(3) $$LAC=\left(LDW\times TECleaves\right)/1000;$$

(4) $$StAC=\left(StDW\times TECstem\right)/1000;$$

(5) TAC=RAC+StAC+LAC.

(6) TI=TDW/TDWT0

(7) $$TF=\left(RAC+LAC+StAC/TEAC\right);$$

(8) $$SAP\left(\%\right)=\left(\left(LAC+StAC\right)/TAC\right)\times 100;$$

(9) $$RAP\left(\%\right)=\left(RAC/TAC\right)\times 100$$

where

RAC, LAC, StAC, and TAC: accumulation of the element in the root, leaves, stems and total plant, respectively;

TI and TF: tolerance index and transfer factor, respectively;

SAP% and RAP%: TE accumulation percentage in the shoot and root system, respectively;

TECroots, TECleaves, and TECstems: concentration of available Cu and Fe (mg kg^−1^) in roots, leaves, and stems, respectively;

RDW, LDW, StDW, and TDW: dry weight (g) of roots, leaves, stems, and the total plant, respectively;

TDWT0: average dry weight of the total plant (g) in the control treatment (T0);

TEAC: concentration (mg) of Cu and Fe available in the 4 dm^3^ of soil.

To calculate iron accumulation in plant organs (RAC, LAC, StAC), the result was divided by 1000, as indicated in Equations (3)–(5), and the result is shown in mg organ^−1^. To calculate copper accumulation, the result was not divided by 1000, and the result is shown in μg organ^−1^. Iron and copper accumulation per plant (TAC) was calculated by adding the accumulations in each organ; therefore, the results are shown in mg plant^−1^ and μg plant^−1^, respectively.

### 2.8. Statistical Analysis

The data were subjected to normality and homogeneity of variance tests using the Shapiro–Wilk and Bartlett tests, respectively, at 5% probability, and, subsequently, subjected to analysis of variance (ANOVA) at 5% probability, as follows: (a) from the significance in the F test, the trend was demonstrated by regression for the variable of concentration of elements in the soil; (b) with one-way ANOVA and with significance in the F test, the comparison of means was performed by Scott–Knott at a 5% significance level [63], for the accumulation variable; (c) with two-way ANOVA and with significance in the F test, the comparison of means was performed by Scott–Knott for the other variables, and the factors analyzed were as follows: TREATMENTS _(0, 50, 150, 250 and 350)_ x TRACE ELEMENTS _(Cu and Fe)_ and, for the partition between shoot and root, TREATMENTS x ORGANS _(shoot and root)_ was also used.

Correlation analyses were performed for each TE. (d) Pearson correlation and (e) network correlation were carried out between the following variables: gas exchange; pigments; biomass; accumulation; accumulation percentage in the shoot and root; and tolerance indices. (f) Principal component analysis (PCA) for each TE was performed between the variables of growth (NL, SD, SL, and TRL), biomass (RDW, LDW, and StDW), accumulation (RAC, LAC, and StAC), accumulation percentage in the shoot and root (SAP and RAP); transfer factor (TF); and tolerance index (TI). The TE factor (Cu and Fe) was used to cluster the PCA. R software, version 4.2.2 (R Foundation for Statistical Computing, Vienna, Austria), was used for data analysis and the creation of graphs [63]. The packages used in R studio are cited and referenced in Supplementary Material S2.

## 3. Results

### 3.1. Availability of Cu and Fe in Soil

**Figure 1:**
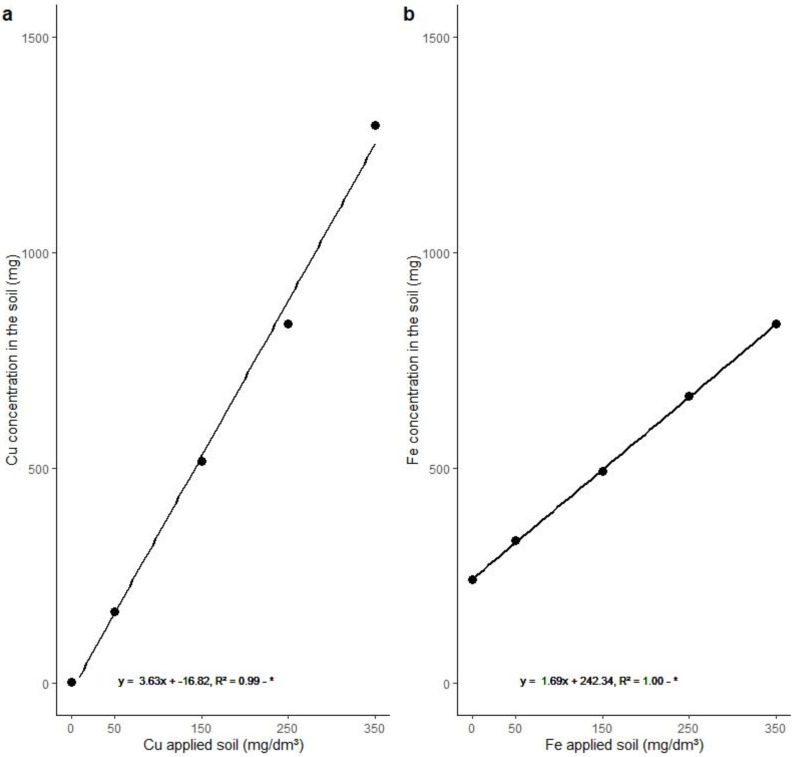
Trace element concentration available (TEAC) in 4 dm^3^ of soil at pH 4.7, thirty days after application of the treatments. (**a**) Cu concentration in the soil (mg); (**b**) Fe concentration in the soil (mg). Number of observations: 30. The line corresponds to the linear adjustment of the significant regression at 5%. *: Significance at the 5% level (*p* < 0.05).

The bioavailability of Cu in the soil (TECsoil) was 0.58, 28.28, 87.80, 141.97, and 220.36 mg Kg^−1^, and the bioavailability of Fe was 40.91, 56.26, 83.74, 113.19, and 141.80 mg Kg^−1^ in T0, T50, T150, T250, and T350, respectively (more information is given in Supplementary Material S3). The TEAC, the concentration of Cu and Fe available in 4 dm^3^ of soil (mg) after the 30-day stabilization period of the treatments in the soil, was calculated. The availability of the elements (TEAC) in the pot (mg) is represented in Figure 1. The TEAC of Cu in the soil was 3.43, 166.31, 516.27, 834.77, and 1295.70 mg, and that of Fe was 240.54, 330.80, 492.40, 665.56, and 855.31 mg in T0, T50, T150, T250, and T350, respectively. Regression analysis showed a tendency to increase the availability of both elements in the soil (4 dm^3^) from the T50 treatment (Figure 1). A list of acronyms and their meanings is available in Supplementary Material S4 for a better understanding of the variables mentioned in the text.

### 3.2. Photosynthetic Pigments

**Figure 2:**
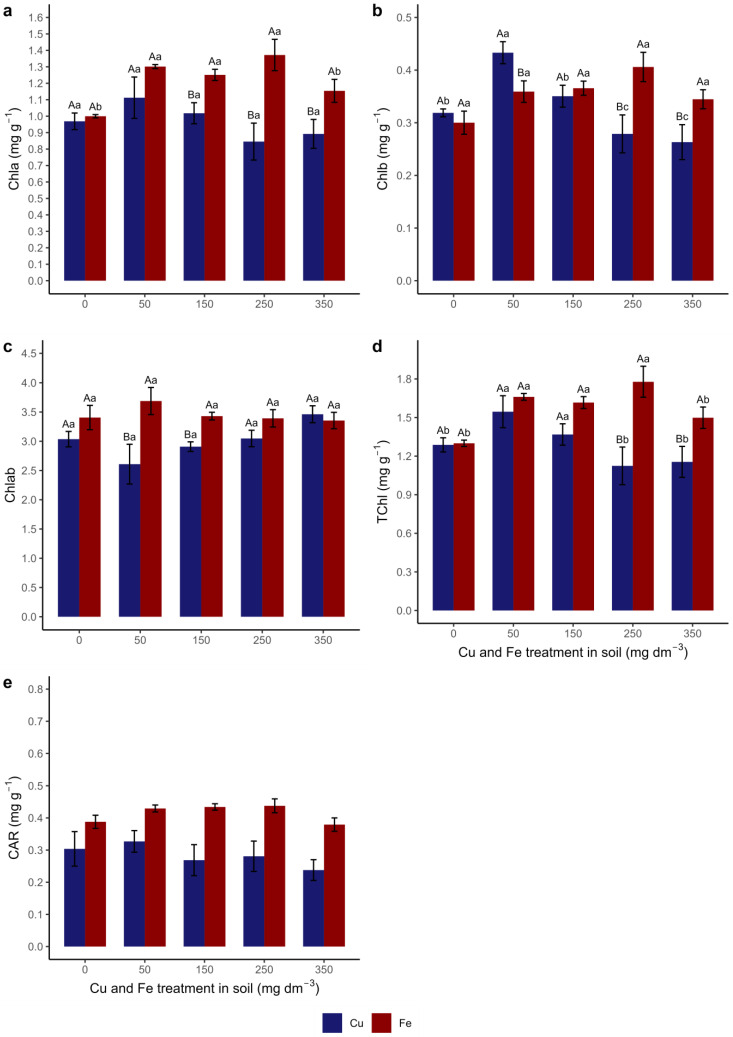
Quantification of photosynthetic pigments in *Canavalia ensiformis* leaves subjected to Cu and Fe treatments in the soil. (**a**) chlorophyll a (Chla); (**b**) chlorophyll b (Chlb); (**c**) chlorophyll a/b ratio (Chlab); (**d**) total chlorophylls (TChl); (**e**) carotenoids (CAR). Number of observations: 60. Mean and standard error are presented, and different letters indicate differences between treatments using the Scott–Knott test (*p* < 0.05), in a double-factorial design. Capital letters indicate a comparison between trace elements in the same treatment, and lower-case letters indicate a comparison between treatments of the same element. No letters indicate no significant difference.

Cu treatments did not affect the plants’ Chla content (Figure 2a). Fe treatments from T50 to T250 showed higher levels of Chla than T0 and T350. The Chla contents were higher in plants grown with Fe from T150 than in Cu treatments.

A reduction in Chlb content was observed (Figure 2b) from T150, mainly in Cu treatments T250 and T350. Chlb contents did not differ between Fe treatments. In T50, plants grown with Cu had a higher content of Chlb than with Fe, with an inversion of this trend occurring in T250 and T350.

The ratio between Chla and Chlb (Chlab) (Figure 2c) did not differ between the treatments of both trace elements. The ratio of chlorophyll Chlab was higher in T50 and T150 treatments with Fe compared to Cu.

The total chlorophyll content (TChl) was higher in T50 and T150 of Cu (Figure 2d). For Fe, the content was higher between T50 and T250. Comparing the contents between both elements, the treatments T250 and T350 showed the highest levels of TChl in cultivations with Fe.

The total carotenoid contents (Figure 2e) did not differ between the treatments with both elements. There was also no interaction between treatments and elements. The plants cultivated with Cu presented approximately 0.28 mg g^−1^ total carotenoids, and plants grown with Fe presented 0.41 mg g^−1^ (T0, T50, T150, T250, and T350).

### 3.3. Gas Exchange

**Figure 3:**
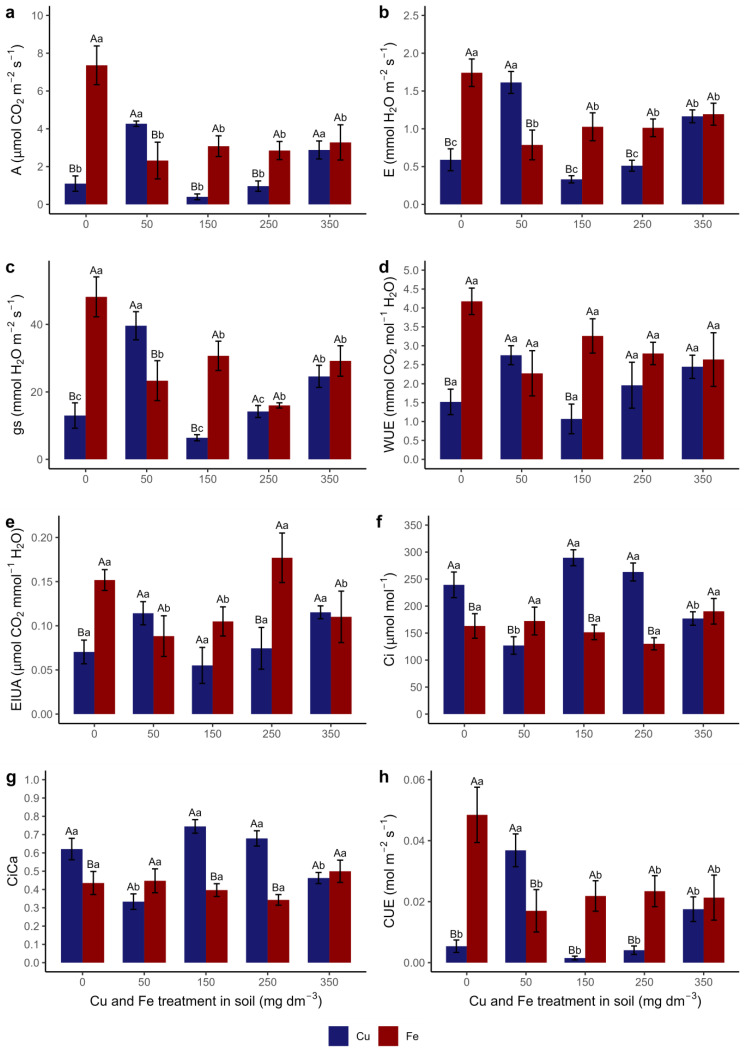
Gas exchange parameters of *Canavalia ensiformis* subjected to Cu and Fe treatments in the soil: (**a**) photosynthetic rate (A); (**b**) transpiration rate (E); (**c**) stomatal conductance (gs); (**d**) water use efficiency (WUE); (**e**) intrinsic water use efficiency (EIUA); (**f**) carbon use efficiency (CUE); (**g**) internal carbon concentration (Ci); and (**h**) internal and external CO_2_ ratio (CiCa). Number of observations: 60. Mean and standard error are presented, and different letters indicate differences between treatments using the Scott–Knott test (*p* < 0.05), in a double-factorial design. Capital letters indicate a comparison between trace elements in the same treatment, and lower-case letters indicate a comparison between treatments with the same element.

The photosynthetic rate (A) (Figure 3a) was higher in T50 and T350 among the other treatments with Cu, while for Fe, only in T0 A was it higher. The highest A in T0, T150, and T250 occurred in plants submitted to Fe and, in T50, it occurred in those submitted to Cu.

The highest transpiration rates (E) (Figure 3b) and stomatal conductance (gs) (Figure 3c) in plants cultivated with Cu occurred in T50, followed by T350 and the other treatments. T0 presented the highest E and gs rates in plants grown with Fe compared to the other treatments. The highest E occurred in plants submitted to Fe in T0, T150, and T250 and in plants submitted to Cu only in T50. Higher gs rates were recorded in plants grown with Fe in T0 and T150 and those grown with Cu in T50.

There was no significant difference between the treatments with each of the elements regarding water use efficiency (WUE) (Figure 3d). Plants cultivated with Fe had higher WUE than those grown with Cu in T0 and T150. There was no difference in intrinsic water use efficiency (IWUA) (Figure 3e) between treatments in plants cultivated with Cu. Plants grown with Fe had higher IWUE in T0 and T250 than in other treatments. When comparing the two elements, plants cultivated with Fe had higher IWUE in T0 and T250.

The T50 of Cu presented higher carbon use efficiency (CUE) (Figure 3f) compared to the other treatments. For Fe, the highest CUE occurred in T0, followed by low CUE in the other treatments. CUE was higher in plants cultivated with Cu only in T50. The other treatments presented higher CUE in plants cultivated with Fe.

The internal carbon (Ci) (Figure 3g) was higher in T0, T150, and T250 in plants cultivated with Cu. There was no difference between treatments of plants grown with Fe. Plants cultivated with Cu presented a higher Ci in T0, T150, and T250, while in T50, the highest Ci value was recorded in plants grown with Fe.

The ratio between internal and external carbon (CiCa) (Figure 3h) was higher in plants cultivated with Cu in T0, T150, and T250. The CiCa ratio did not differ between Fe treatments. Plants grown with Cu presented a higher CiCa ratio in T0, T150, and T250, while in T50, the highest value was recorded in plants cultivated with Fe.

It can also be observed that in T350, of all the gas exchange variables analyzed, there was no difference between Cu and Fe; that is, the species responds similarly to the concentration of both elements in T350.

### 3.4. Growth Parameters

**Figure 4:**
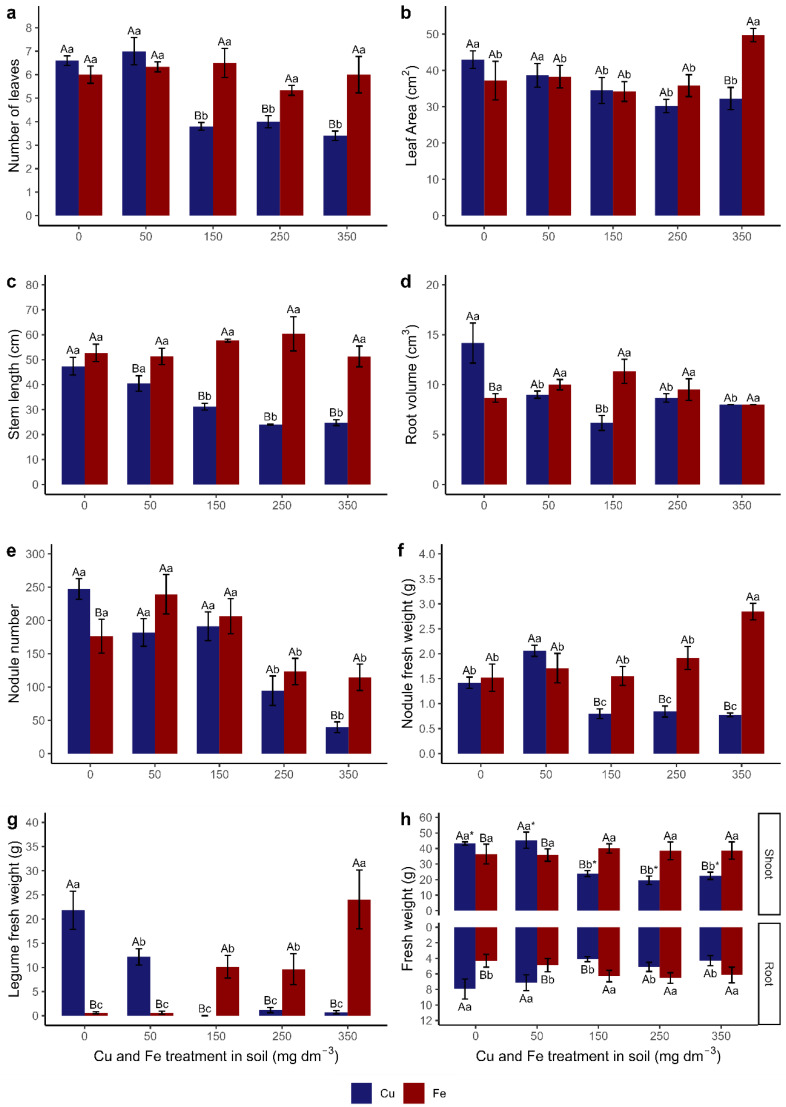
Growth parameters of *Canavalia ensiformis* subjected to Cu and Fe treatments in the soil: (**a**) number of leaves; (**b**) leaf area (cm^2^); (**c**) stem length (cm); (**d**) root volume (cm^3^); (**e**) nodule number; (**f**) nodule fresh weight (**g**); legume fresh weight (**g**); and (**h**) fresh weight partitioning between the shoot and the root (**g**). Number of observations: 60. Mean and standard error are presented, and different letters indicate differences between treatments using the Scott–Knott test (*p* < 0.05), in a double-factorial design. Capital letters indicate a comparison between trace elements in the same treatment, and lower-case letters indicate a comparison between treatments of the same element. * indicates that the respective organ has the largest partition difference.

**Figure 5:**
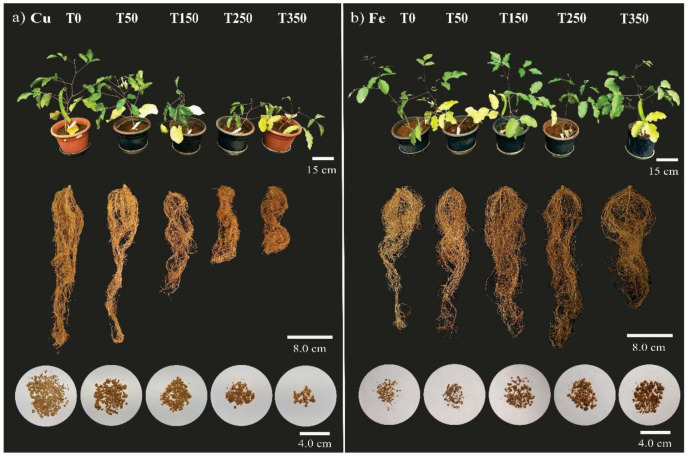
Shoot and root system and nodules of *Canavalia ensiformis* subjected to concentrations of Cu (**a**) and Fe (**b**). The treatments added, in addition to the control, were 50; 150; 250, and 350 mg dm^−3^ of soil in a greenhouse for 90 days.

The number of leaves (Figure 4a and Figure 5) between treatments in plants cultivated with Cu decreased from T150, while there was no difference between treatments in plants cultivated with Fe. For T150, plants grown with Fe presented more leaves/plants than those grown with Cu.

The leaf area (Figure 4b and Figure 5) of the plants cultivated with Cu decreased from T150, while the T350 plants presented the highest leaf area among treatments with Fe. Between treatments, there was a difference in leaf area only in T350; plants grown with Fe showed a greater leaf area than plants treated with Cu.

The stem length (Figure 4c and Figure 5) of plants cultivated with Cu was shorter from T150, while there was no significant difference between treatments for Fe. The stem length was similar in T0 for both elements and longer in T50 plants grown with Fe than those grown with Cu.

The root volume (Figure 4d and Figure 5) of plants cultivated with Cu between T50 and T350 was lower than in T0, while the root volume of plants cultivated with Fe did not differ between treatments. Plants grown in T0 with Cu had a higher root volume than those grown with Fe, while the reverse occurred in T150. The other treatments did not differ between Cu and Fe.

The number of nodules (Figure 4e and Figure 5) decreased in T250 and T350 for both elements. Plants cultivated with Fe presented more nodules than plants with Cu between T50 and T350. The fresh weight of nodules (Figure 4f) was higher in T50, followed by T0 and the other Cu treatments. In T350, plants grown with Fe showed the highest weight of nodules, followed by the other treatments. From T150 onwards, plants grown with Fe presented a higher fresh weight of nodules than those grown with Cu.

The plants cultivated with Cu had a higher fresh pod weight in T0 (21 g) (Figure 4g). The fresh mass in T50 (12 g) and the other treatments was less than 1.5 g (Figure 4g). The reverse occurred for plants grown with Fe. The plants did not produce pods in T0 and T50, but pod production was found in T150, T250, and T350. The highest fresh weight of pods was found in T350. Plants cultivated with Cu had a higher fresh pod weight in T0 and T50 than plants grown with Fe. Between T150 and T350, the fresh pod weight was higher in plants cultivated with Fe.

The fresh weight partition between the shoot (leaves + stem) and root system was analyzed (Figure 4h and Figure 5). The fresh weight of the shoots of plants cultivated with Cu was higher in T0 and T50, followed by a decrease in mass in the other treatments. Plants grown with Fe showed no difference in the fresh weight of the shoots between treatments. In T0 and T50, plants grown with Cu presented a higher shoot weight, while the reverse occurred with plants grown with Fe in T150, T250, and T350, which presented an approximately 50% higher fresh shoot weight. The fresh root mass (Figure 4h and Figure 5) of plants cultivated with Cu decreased from T150, while there was an increase in mass for plants grown with Fe. Plants cultivated with Cu had a higher fresh root mass in T0 and T50, while plants cultivated with Fe presented a higher root mass in T150. There was no difference between the trace elements in T250 and T350.

In the comparison of fresh weight partition (shoots and roots) (Figure 4h), we observed more shoot biomass (represented in the graph by “*”) than root biomass in plants cultivated with Cu. There was no difference in fresh weight partition for plants grown with Fe.

### 3.5. Dry Biomass and Phytoremediation Indices

**Figure 6:**
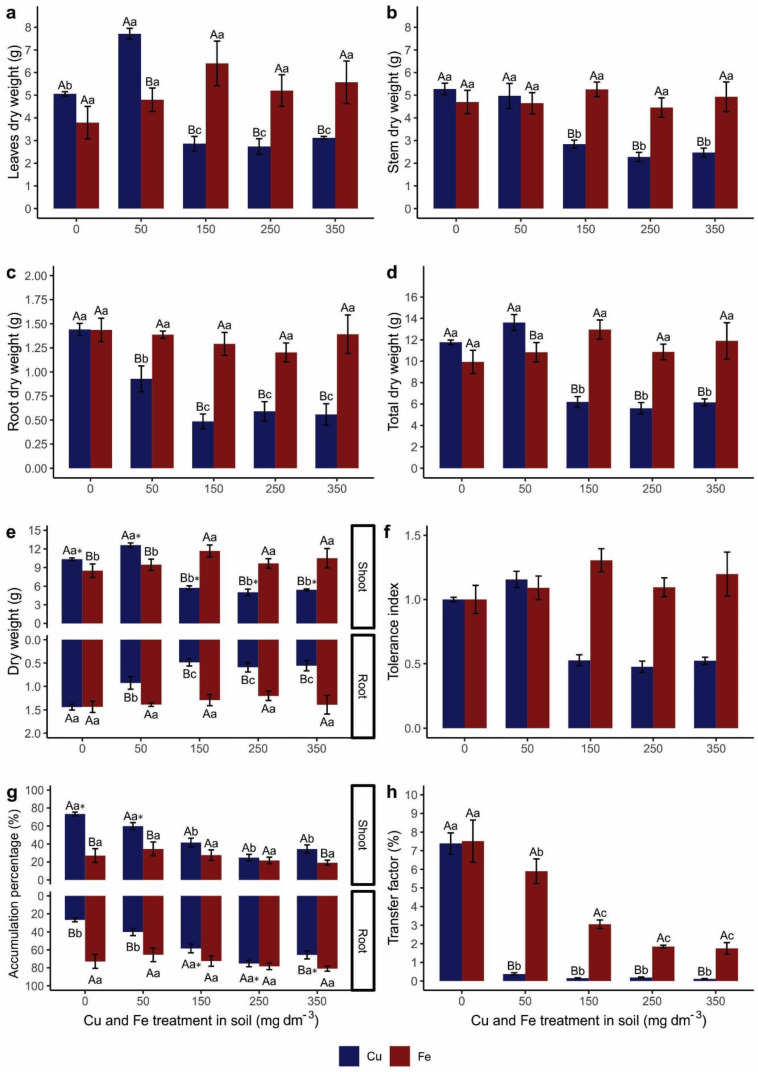
Dry weight and phytoremediation indexes of *Canavalia ensiformis* subjected to Cu and Fe treatments in the soil. (**a**) Leaf dry weight (g); (**b**) stem dry weight (g); (**c**) root dry weight (g); (**d**) total plant dry weight (g); (**e**) partitioning of dry weight between the shoot and the root (g); (**f**) tolerance index (qualitative data); (**g**) percentage of PTEs accumulation between the root (RAP) and shoot (SAP) organs (%); (**h**) transfer factor. Number of observations: 60. Mean and standard error are presented, and different letters indicate differences between treatments using the Scott–Knott test (*p* < 0.05), in a double-factorial design. Capital letters indicate a comparison between trace elements in the same treatment, and lower-case letters indicate a comparison between treatments with the same element. * indicates that the respective organ has the largest partition difference.

Plants grown with Cu produced more leaf biomass (dry weight) (Figure 5 and Figure 6a) in T50 and T0, followed by a decrease of more than 50% in the other treatments. Plants grown with Fe showed no differences in leaf biomass between treatments. In T50, the leaf biomass was higher in plants cultivated with Cu than in those cultivated with Fe. Between T150 and T350, the leaf biomass was higher in plants cultivated with Fe.

The stem biomass (Figure 6b) of plants cultivated with Cu was higher between T0 and T50, with biomass reduction in T150. No differences in stem biomass were detected between Fe treatments. When comparing both elements, there was no difference in T0 or T50, while the stem biomass in plants grown with Fe was higher than in Cu between T150 and T350.

The root biomass (Figure 5 and Figure 6c) of the plants cultivated with Cu was higher in T0, followed by T50. The other treatments had a decrease in biomass of 150%. Plants grown with Fe showed no differences in root biomass between treatments. When comparing both elements, there was no difference in T0 or T50, while the root biomass in plants grown with Fe was higher than in those grown with Cu between T150 and T350.

Plants grown with Cu produced higher total biomass (leaves, stems, and roots) (Figure 6d) at T0 and T50, followed by a decrease of more than 50% in the other treatments. Plants grown with Fe showed no differences in total biomass between treatments. There was no difference in T0 when comparing both elements. In T50, the total biomass was higher in plants cultivated with Cu than in those cultivated with Fe. Between T150 and T350, the total biomass was higher in plants cultivated with Fe.

The biomass partition between the shoot (leaves + stem) and root system was analyzed (Figure 6e). The shoot biomass of plants grown with Cu was higher in T0 and T50, with a decrease in biomass in the other treatments. Plants grown with Fe had higher shoot biomass between T150 and T350. The other treatments presented lower biomass. The shoot biomass was higher in plants grown with Cu than in those grown with Fe in T0 and T50, while plants grown with Fe had higher shoot biomass in the other treatments. The biomass partition of the root system was previously described (Figure 6c).

When comparing the dry biomass partition (shoots and roots) between the organs (Figure 6e), plants grown with Cu had higher shoot biomass (represented in the graph by “*”) than root biomass. There was no difference in dry biomass partition between organs in plants cultivated with Fe.

*Canavalia ensiformis* was tolerant to all Fe treatments evaluated (TI ≥ 5.0); however, for Cu, from T150 onwards, the TI was less than 0.5. Therefore, *C. ensiformis* was not tolerant to T150, T250, and T350 Cu treatments (TI ≤ 5.0) (Figure 6f).

We estimated how much (%) of the element was translocated and accumulated to the shoot, called the shoot accumulation percentage (SAP), and how much (%) was absorbed and accumulated in the root, which we call the root accumulation percentage (RAP) (Figure 6g). The T0 and T50 plants translocated more Cu to the shoot than the other treatments: the SAP was 73.32 and 59.79%, respectively, for an average of 66.56%. There was no significant difference in SAP between Fe treatments (on average, 25.99%). Between the two elements, the percentage of Cu accumulation in the shoot was 46.24, 25.28, and 15.27% higher than Fe in T0, T50, and T350, respectively; that is, the plants translocated more Cu (55.84%) to the shoot than Fe (26.91%).

The plants of T150, T250, and T350 presented the highest percentage of Cu accumulation in the root than the other treatments. The RAP was 58.40, 75.19, and 65.60% of Cu. There was no significant difference in RAP between Fe treatments. Between the two elements, the RAP of Fe was 46.24, 25.28, and 15.67% higher than Cu in T0, T50, and T350, respectively (Figure 6g).

Calculating the mean SAP and RAP of all treatments, we observed that 46.79% of Cu and 25.99% of Fe were translocated and accumulated by the shoot of the plants. The other 53.21% of Cu and 74.01% of Fe were accumulated in the roots. Thus, both elements were absorbed and accumulated in a higher percentage in the root (RAP). Cu was the element most translocated and accumulated in the shoot of the plants (SAP) (Figure 6g).

The transfer factor (Figure 6h) refers to an element’s transfer/transport (%) from the soil to the plant. The transfer factor between T50 and T350 for Cu was less than 1%. For Fe, the transfer factor in T50 was approximately 6%, and for T150, it was less than 3%. In general, Fe transfer was higher than Cu transfer.

### 3.6. Accumulation of Cu and Fe

**Figure 7:**
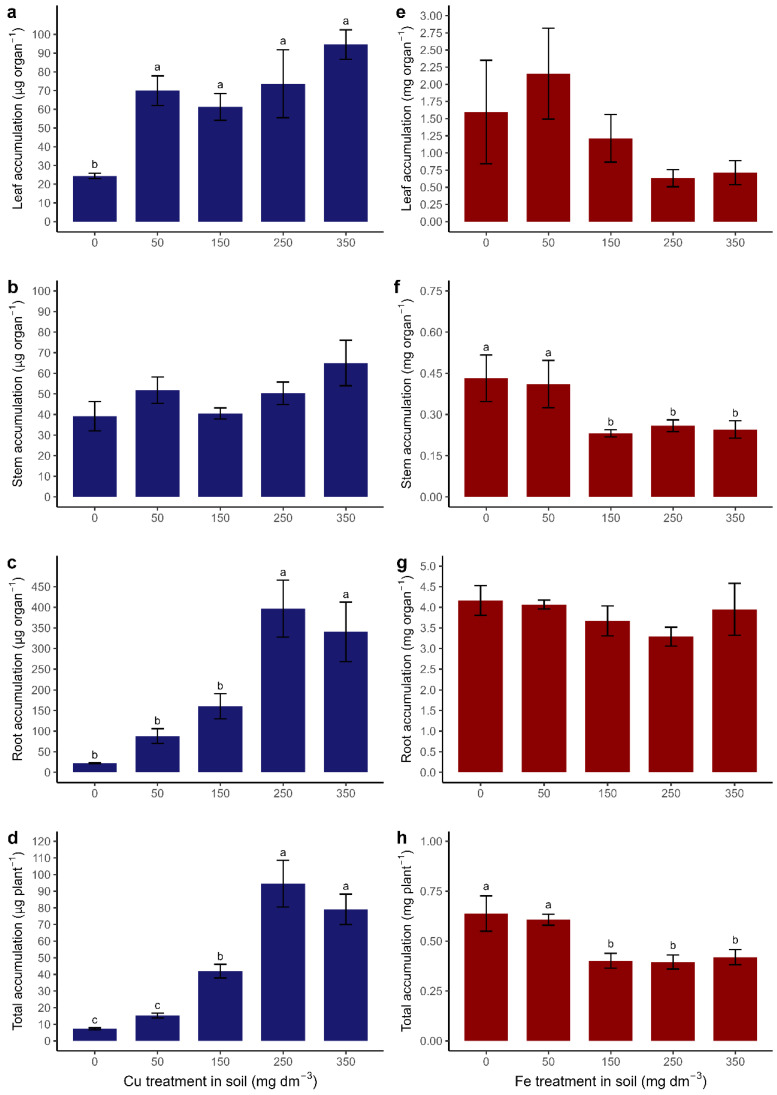
PTE accumulation in the organs of *Canavalia ensiformis* submitted to Cu and Fe treatments in the soil. (**a**) and (**e**) PTE accumulation in the leaf; (**b**) and (**f**) PTE accumulation in the stem; (**c**) and (**g**) PTE accumulation in the roots; (**d**) and (**h**) PTE accumulation in the total plant. (**a**) to (**c**) Cu accumulation in µg organ^−1^; (**d**) Cu accumulation per plant in µg plant^−1^; (**e**) to g) Fe accumulation in mg organ^−1^; (**h**) Fe accumulation per plant in mg plant^−1^. Number of observations: 30. Mean and standard error are presented, and different letters indicate differences between treatments using the Scott–Knott test (*p* < 0.05). Absence of letters indicates no differences.

Cu accumulation in leaves (Figure 7a) from T50 to T350 was higher (±74.84 µg organ^−1^) than in T0 (±24.39 µg organ^−1^). Cu accumulation in the stem (Figure 7b) did not differ between treatments (±49.33 µg organ^−1^). Cu accumulation in the root (Figure 7c) was higher in T250 and T350 (±368.63 µg organ^−1^), with low accumulation in the other treatments (±90.23 µg organ^−1^). The total accumulation (leaves, stems, and roots) (Figure 7d) of Cu was higher for T250 and T350 (±86.8 µg plant^−1^), followed by T150 (±41.95 µg plant^−1^) and T0 (±7.31 µg plant^−1^).

Fe accumulation in the leaves (Figure 7e) did not differ between treatments (±1.25 mg organ^−1^). The accumulation of Fe in the stem (Figure 7f) was higher in T0 and T50 (±0.42 mg organ^−1^) than in the other treatments (±0.24 mg organ^−1^). Fe accumulation in the root system (Figure 7g) did not differ between treatments (±3.82 mg organ^−1^). Total Fe accumulation (Figure 7h) was highest for T0 and T50 (±0.61 mg plant^−1^), followed by the other treatments (±0.40 mg plant^−1^).

### 3.7. Multivariate Analysis

**Figure 8:**
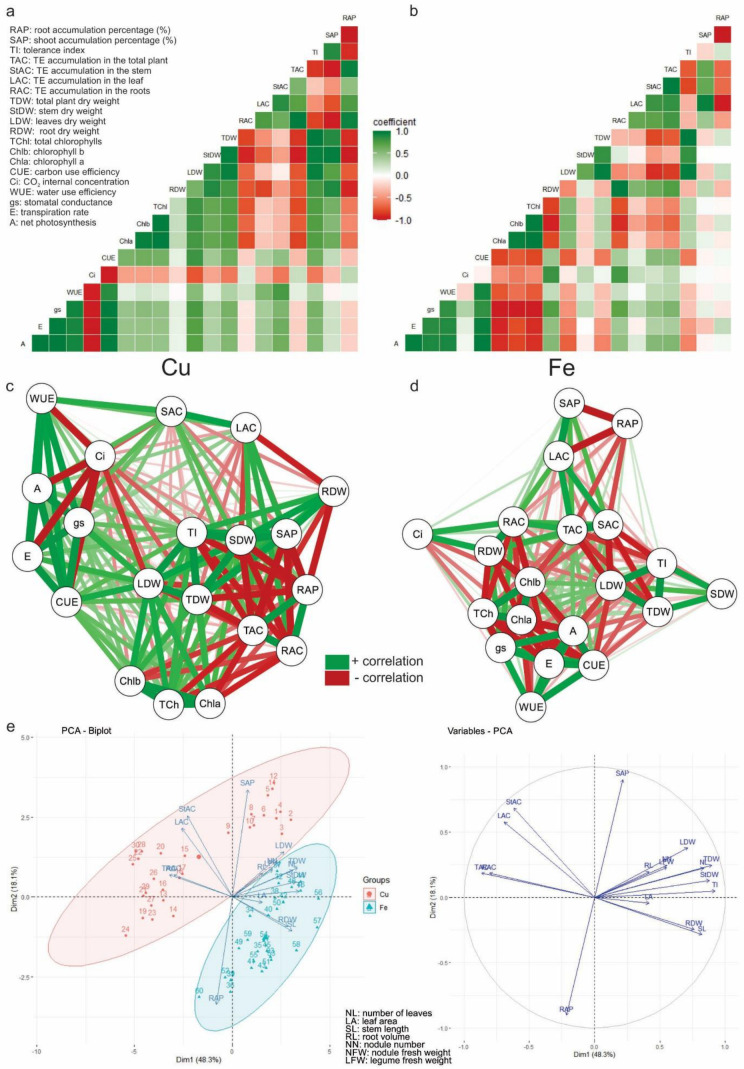
Multivariate analysis: correlation and principal component analysis (PCA). Correlation analysis was performed with the variables pigments, gas exchange, biomass, accumulation, tolerance index and percentage of accumulation: (**a**,**b**): Pearson correlation (r); (**c**,**d**) network correlation. The left and right side show the correlations of *Canavalia ensiformis* with Cu and Fe, respectively; (**e**) principal component analysis—PCA (PCA-biplot and PCA-variables). Pearson correlation (r): coefficient = 1 indicates a positive correlation between the variables; coefficient = −1 indicates negative correlation; coefficient = ±0.5 indicates a moderate correlation; coefficient = 0 indicates that the variables do not depend linearly on each other. Network correlation: each variable is a node, and each correlation is a connecting edge/line. Closer, correlated relationships have a thicker edge/connection, and the more intense the color, the stronger the relationship between them (R ≥ 0.7, and the significance level is *p* < 0.05). In both correlations, intense green indicates a strong positive correlation (as the value of one variable increases, so does the value of the other variable); deep red means a strong negative correlation (as the value of one variable increases, the value of the other decreases). The principal component analysis biplot was performed with the growth variables; biomass; index of tolerance; and percentage of accumulation. The acronyms of the variables analyzed were as follows: A: photosynthetic rate; E: transpiration rate; gs: stomatal conductance; WUE: water use efficiency; Ci: CO_2_ internal concentration; CUE: carbon use efficiency; Chla: chlorophyll a; Chlb: chlorophyll b; TChl: total chlorophylls; RDW: root dry weight; LDW: dry weight of leaves; StDW: stem dry weight; TDW: total biomass; RAC: root accumulation; LAC: accumulation in leaves; StAC: stem accumulation; TAC: accumulation in total biomass; TI: tolerance index; SAP: shoot accumulation percentage; RAP: root accumulation percentage; NL: number of leaves; LA: leaf area; SL: stem length; RL: root volume; NN: number of nodules; NFW: nodule fresh weight; LFW: fresh pod weight. Number of observations: 30.

The correlation matrix (Pearson and network) (Figure 8) allowed us to identify associations and relationships between the variable’s gas exchange, pigments, growth, biomass, accumulation, tolerance, percentage of accumulation, and element treatments separately. In *C. ensiformis* cultivated under Cu treatments (Figure 8a,c), the observed relationships between A, E, gs, WUE, and CUE; Chla, Chlb, and TChl; LDW, StDW, TDW, and TI; LAC and StAC; TI and RAP; SAP, StDW, and TDW; and TAC, RAC, and RAP are positive correlations. The relationships between Ci + A, E, gs, WUE, and CUE; RAC + Chla, TChl, StDW, TDW, SAP, and TI; RDW + LAC and RAP; RAP + SAP, StDW, and TDW; TI + TAC; and TAC + TDW, StDW, Chla, TChl, and SAP are negative correlations.

In *C. ensiformis* cultivated under Fe treatments (Figure 8b,d), the observed relationships between A, E, gs, WUE, and CUE; Chla, Chlb, and TChl; Ci, RDW, and RAC; StDW, TDW, and TI; LDW, TDW, and TI; TAC, StAC, LAC, RAC; and SAP and LAC are positive correlations. The relationships between Chla + CUE, A, E, and gs; Chlb + gs, RDW, RAC, E, and A; TChl + RDW and RAC; TDW + TAC and StAC; RAP + SAP and LAC; and TI + TAC, StAC are negative correlations.

The principal component analysis of the variables of growth, biomass, accumulation tolerance index, and translocation was carried out to group these variables according to the behavior of the variation in their characteristics in a specific data set: Cu or Fe. The results indicated 66.4% variance (Figure 8e). According to the results, the interrelationships among all variables for the Cu element comprise RAC, TAC, LAC, StAC, SAP, LDW, NFW, LFW, NN, and RL. For Fe, the interrelationships comprise RAP, TI, RDW, StDW, TDW, number of leaves (NL), SL, and leaf area (LA).

## 4. Discussion

Copper and iron ions are initially absorbed similarly between dicotyledons and non-grassy monocotyledons [64]. They are accumulated by the roots and translocated to other parts of the plant. Both PTEs were accumulated mainly by the roots, except for the availability characteristics of each element in the rhizosphere. This accumulation was responsible for the species’ different physiological and growth responses to each element since the roots are organs sensitive to perception and stress signaling [65].

The results indicate that Cu treatments at concentrations ranging from 150 to 350 mg dm^−3^ were more detrimental to the growth of *C. ensiformis* compared to the same iron treatments.

### 4.1. Copper

Copper treatments (>T150) negatively affected the volume and biomass of the root system. The number and fresh weight ratio of nodules were also affected (Figure 4, Figure 5 and Figure 6). Excess Cu prevents root growth. The plants presented the following characteristics: decreased elongation of the primary root; lateral roots; and proliferation of root hairs, consequently impairing growth and shoot biomass production [66,67,68]. It was verified that the root system of plants cultivated with Cu presented the highest percentage of Cu accumulation (RAP) and consequently affected the accumulation of root biomass, which in turn directly influenced the length, dry weight of the stem, and the total biomass of the plant (negative correlation) (Figure 8), as occurred in *Medicago sativa* [69], *Calopogonium mucunoides* [68], *Stizolobium aterrimum* [70], and *C. ensiformis* treated and not treated with arbuscular mycorrhizal fungi [26].

The excess Cu impaired both root and shoot biomass. There is a relationship between shoot biomass and the number of nodules that may explain this reduction: shoot biomass influences the metabolic activity of the nodules. It interferes with the FBN of legumes [68,70]. The leaf area determines the efficiency of the photosynthetic organism and the ability to transform light energy into chemical energy to produce photoassimilates/carbohydrates, which are transported to the nodules and used as an energy source for the growth and respiration of symbiotic bacteria that perform FBN and for mycorrhizal fungi [71,72,73,74].

The accumulation of plant biomass (TDW) is directly related to the tolerance index (TI) of the species to Cu. Under these conditions, *C. ensiformis* proved not to be tolerant to copper since the tolerance index is less than 0.5 from T150. Treatments above 100 mg dm^−3^ of Cu caused a reduction in plant biomass and consequently may affect plant tolerance, as in the case of *Stizolobium aterrimum* [70]. In addition, there is no carbon allocation to the shoot or root (Figure 6 and Figure 8). The higher the percentage of Cu accumulation at the root, thus avoiding translocation and accumulation to the shoot (SAP), the more it can interfere with the plant’s total biomass accumulation and change the species’ tolerance degree (Figure 8). The higher the percentage of Cu accumulation at the root, the lower the percentage of accumulation in the shoot (negative correlation) and the more resources invested in shoot biomass (partition) (Figure 6 and Figure 8).

Cu accumulation moderately affected chlorophyll levels (a, b, and total), presenting a decrease in Chlb and TChl only in T250 and T350 (Figure 2). While Cu is part of the composition of plastocyanin, a membrane protein present in the thylakoids of chloroplasts, responsible for the photosynthetic reactions of electron transport, and participates in the formation/synthesis of chlorophyll [75], its excess can affect synthesis or degrade chlorophylls, as demonstrated in this study and others [34,47,76,77].

The relationship between chlorophylls and Cu accumulation (RAC and TAC) is likely related to the changes observed in gas exchange since the behavior of Chla and Chlb levels throughout the treatments is similar to that of A, gs, E, CUE, and WUE (moderate positive correlation) (Figure 2, Figure 3 and Figure 8) as reported by other authors [78,79]. The rate of net photosynthesis (assimilation of CO_2_), stomatal conductance, transpiration, and carbon and water use efficiency are proportionally correlated (positive correlation). However, they are inversely proportional to the internal concentration of CO_2_ (negative correlation); that is, when A, gs, E, CUE, and WUE decrease, Ci increases (Figure 3 and Figure 8).

In addition to gas exchange, decreased levels of Chlb and TChl also influenced the NL and leaf biomass (LDW) (moderate positive correlation), in addition to LA (Figure 4, Figure 5 and Figure 6 and Figure 8), as observed by other authors [67,80]. Other important relationships include the positive correlations between LDW and gs and CUE and Cu accumulation in the stem (StAC) and WUE. Because the accumulation of Cu in the stem was insignificant, it did not interfere with the harvested biomass, water use ratio, or low accumulation in the leaves (LAC), which was fundamental for the carbon balance (CUE). Even having decreased with higher treatments, LDW was important for the plants in completing their life cycle. In addition, stomatal conductance is LDW-dependent since gs depends on the number of stomata per unit leaf area (stomatal density) and the degree of stomatal opening.

### 4.2. Iron

The availability and accumulation of iron (RAC and RAP) in the roots did not impair growth and root biomass. The number of nodules decreased in T250 and T350, but in compensation, the fresh weight of nodules increased in T350. Fe is crucial for biological nitrogen fixation in leguminous plants, since it is present in some proteins and enzymes such as leghemoglobin and nitrogenase [80,81,82,83]; therefore, excess Fe may have caused a restriction in the formation of new nodules and the growth of pre-existing nodules (Figure 4, Figure 5 and Figure 6).

Shoot biomass was also not negatively affected by Fe. On the contrary, the leaf area in T350 increased. Shoot biomass increased from T150 to T350 (Figure 4, Figure 5 and Figure 6). Shoot biomass—LDW, StDW, and TDW (Figure 8b,d)—was responsible for the tolerance index (TI), with positive correlation, in addition to other factors such as SL, LA, and NL, as demonstrated by the principal component analysis (Figure 8e).

Both RDW and RAC are inversely proportional to chlorophyll content. The higher the biomass and Fe accumulation in the root, the less chlorophyll accumulated, especially Chla and TChl, explaining the high levels of Chlab for T350 (Figure 2, Figure 7 and Figure 8). Chlorophyll levels are inversely proportional (negative correlation) to gas exchange parameters (Figure 8b,d). There are lower gs, A, and E values in treatments with higher chlorophyll levels. Fe is essential for chlorophyll biosynthesis, so the balance between the import and export of Fe to and from the chloroplast is extremely important for adequate photosynthetic activity productivity [84]. This balance depends on the accumulation of Fe in the roots and how much is or is not translocated to the shoot. Fe homeostasis represents an essential process for optimal plant productivity [84].

The internal concentration of CO_2_ is directly related to the accumulation of root biomass, which was responsible for the accumulation of Fe in the root system (Figure 8b,d). The more Fe is accumulated in the roots, the more it will accumulate in the shoot and the total biomass (positive correlation). In addition to Ci, gs presents a positive and moderate relationship with RDW and RAC and a weak relationship with the other gas exchange parameters, indicating that Fe accumulation does not directly interfere with gas exchange (Figure 8b,d).

The percentage of Fe accumulation in the root (RAP) presents a negative correlation with the shoot accumulation percentage (SAP) and Fe accumulation in the leaves (LAC); that is, the more Fe is accumulated in the root, the less Fe is translocated to the shoot and in the leaves (Figure 8b,d), characterizing a defense mechanism, since most of the Fe is stored in the apoplast of the roots than in the leaves, in addition, other organelles such as vacuoles and plastids also play a fundamental role in the intracellular compartmentalization of Fe [85,86].

### 4.3. Common Responses to Cu and Fe

The root system is the organ with the highest percentage of accumulation for both elements. The accumulation of both elements influenced the chlorophyll levels (they were inversely proportional); that is, the more Cu and Fe accumulated, the lower the levels of chlorophyll. Biomass accumulation (TDW) is proportional to the chlorophyll levels of the leaves. Consequently, it is also proportional to the tolerance index for both elements. In turn, IT is inversely proportional to the accumulation of PTEs.

Both elements’ shoot accumulation percentage (SAP) is inversely proportional to the percentage of accumulation in the root (RAP). In other words, the greater the accumulation of the elements in the root, the less they will be translocated to the shoot of the plants, configuring an important defense mechanism [61] to avoid high concentrations of these elements in the leaves and thus protect the photosynthesizing apparatus.

### 4.4. Different Responses

Chlorophyll levels influenced the gas exchange parameters differently in response to each PTE. Cu accumulation triggered a positive dependence relationship between chlorophyll levels and gas exchange, unlike the response to Fe accumulation.

Chlorophyll levels and carbon assimilation (A) decrease and internal carbon (Ci) increases as Cu accumulation rises. Chlorophyll levels, assimilation, and internal carbon decrease with the accumulation of Fe.

*Canavalia ensiformis* subjected to copper preferred biomass accumulation at low doses (T0–T50) and Cu accumulation at high doses (T150–T350), while *C. ensiformis*, when subjected to Fe, maintained constant biomass accumulation even at high iron doses (T150–T350).

### 4.5. Limitations and Future Perspectives

The contrasting behavior of Cu and Fe in the soil system can be attributed to their distinct speciation pathways under acidic conditions. Cu remains predominantly in its ionic form (Cu^2+^), which enhances its solubility and mobility, increasing the likelihood of phytotoxic effects [87,88]. Fe, on the other hand, is more prone to interactions with soil constituents such as oxides and organic matter, which can lead to its stabilization through precipitation or complexation [89,90]. These mechanisms help explain the discrepancy between environmental availability and plant accumulation observed in *Canavalia ensiformis*, where Fe uptake was more pronounced despite its lower bioavailable fraction.

The acidic pH range recorded in the experimental soil (pH 4.7) played a central role in shaping the geochemical dynamics of PTEs. Under such conditions, Cu remains highly reactive and accessible to plant roots, while Fe may transition into less soluble forms or become sequestered by colloidal particles and organic ligands. This behavior, the possible Fe complexation, and its low availability align with findings by Batista et al. [45] and da Silva et al. [88], who demonstrated that alkaline tailings (pH 6.7–8.0) suppress the availability of PTE such as Fe, whereas acidification below pH 6.0 can trigger the release of adsorbed PTEs into the soil solution, posing significant environmental risks [88,91,92,93]. In our study, the acidic environment likely intensified the environmental presence of Cu, contributing to its elevated toxicity relative to Fe.

Soil physicochemical properties further modulate the fate of Cu and Fe. The moderate organic matter content (16 g dm^−3^), low cation exchange capacity (CEC = 2.61 cmolc dm^−3^), and predominantly sandy texture suggest limited retention of cationic species. These conditions favor Cu persistence in the soil solution, while Fe may have been partially immobilized through complexation with organic ligands or precipitation as hydroxides [87,94]. Such interactions reinforce the notion that Cu toxicity in this system is a consequence of both its chemical form and the restricted buffering potential of the soil.

In addition to assessing tolerance, growth, gas exchange, biomass, and accumulation of PTEs in each plant species, it is essential to investigate nitrogen and carbon metabolism; enzymatic and non-enzymatic antioxidant pathways; membrane integrity; plant nutrition; the expression of metal transporters related to the uptake and compartmentalization of PTEs; and ultrastructural anatomical changes in roots, stems, and leaves caused by PTEs [47,51,68,70,77,95,96]. These variables are crucial for a deeper understanding of the biochemical and structural mechanisms involved. Although these stress-related markers were not addressed in the present study, our findings provide a valuable basis for new insights, and the phytoremediation potential of *Canavalia ensiformis* can be further explored in future research.

Given the complexity of these interactions, it is essential to emphasize that the dynamics of PTE availability and toxicity must be evaluated on a case-by-case basis. The relationship between soil properties, potentially toxic elements speciation, and plant species-specific uptake mechanisms is highly contextual and cannot be generalized. Future studies should explicitly consider the triadic system of soil + PTE + species, as each component plays a decisive role in determining environmental risk and physiological impact.

## 5. Conclusions

The dynamics of the availability of potentially toxic elements varies between Cu and Fe and depends on soil conditions and types. This dynamic must be investigated on a case-by-case basis, as must be the relationship of a particular species with this system: soil + PTE + species. Due to soil conditions, copper became more environmentally available in the soil than iron. However, the absorption and accumulation of iron in plants was more significant than the accumulation of copper, which may be related to the element’s toxicity.

The percentage of accumulation of both PTEs in the roots was high. Consequently, the translocation to the shoot was low. *C. ensiformis* showed potential for Cu and Fe phytostabilization in the soil under the tested conditions (soil + PTE + species).

Each element caused different plant stress levels, triggering different physiological responses in *Canavalia ensiformis* to resist the damage caused. While *Canavalia ensiformis* was tolerant to the Fe concentrations tested (up to 141.80 mg Kg^−1^), the species was not tolerant to concentrations exceeding 87.80 mg Kg^−1^ of Cu in the soil (T150). Under high Cu doses (T150–T350), *C. ensiformis* preferred copper accumulation over biomass accumulation. However, under excessive iron in the soil, *C. ensiformis* preferred biomass accumulation over iron accumulation in its tissues (T0–T350).

Excess copper in the soil affected root development, nodulation, growth of the shoot, biomass accumulation, and pod production, demonstrating signs of toxicity in treatments in 150, 250, and 350 mg Cu.dm^−3^ of soil. Higher available concentrations of Cu in the soil are expected to be lethal to plant development or compromise the growth and establishment of *C. ensiformis*.

The accumulation of iron was not as harmful as copper for *C. ensiformis*. The excess iron affected pigments and gas exchange but not enough to affect growth and biomass accumulation. This paper contributes significantly to the knowledge of physiological responses to stresses by copper and iron throughout the plant cycle, primarily by iron, which is rarely found in the literature in an environmentally available form in the soil or combined with other PTEs.

The inhibition of, or a decrease in, chlorophyll synthesis alters the plant’s photosynthetic efficiency, decreases biomass production, as mentioned above, and consequently influences the tolerance potential of the species. Thus, photosynthesizing pigments and gas exchange responses are essential in assessing the tolerance of plant species exposed to each element.

## Figures and Tables

**Table 1:** Physicochemical characterization of the soil; the soil layer 0–40 cm deep was used (Oxisol).

Layer 0–40 cm	Soil Chemical Characterization
5.3	pH
50	Base saturation, V%
1.31 cmolc.dm^−3^	Sum of bases, SB
0.7 cmolc.dm^−3^	Calcium, Ca^2+^
0.01 cmolc.dm^−3^	Exchangeable potassium, K^+^
0.06 cmolc.dm^−3^	Exchangeable magnesium, Mg^2+^
1.3 cmolc.dm^−3^	Potential acidity, H^+^ + Al^3+^
0.00 cmolc.dm^−3^	Aluminum, Al^3^
1.00 mg dm^−3^	Phosphorus resin, P
16.00 g dm^−3^	Organic matter, OM
2.61 cmolc.dm^−3^	Cation exchange capacity, CEC
	**Micronutrient Analysis**
5.50 mg dm^−3^	DTPA manganese, Mn
16.00 mg dm^−3^	DTPA iron, Fe
0.50 mg dm^−3^	DTPA copper, Cu
0.20 mg dm^−3^	DTPA zinc, Zn
0.06 mg dm^−3^	Boron, B
**Physical characterization of the soil**	
134 g kg^−1^	Clay
855 g kg^−1^	Sand
12 g kg^−1^	Silt
1.47 kg dm^−3^	Density

## Data Availability

Data are available from the corresponding author on reasonable request.

## Supplementary Materials

The following supporting information can be downloaded at https://www.mdpi.com/article/10.3390/metabo15110706/s1, Supplementary Material S1: Reference concentrations for copper and iron in soil mg kg^−1^; Supplementary Material S2: Statistical packages used in R software [97,98,99,100,101,102,103,104,105,106,107,108,109,110,111,112,113,114,115,116,117]; Supplementary Material S3: Table S1: Descriptive statistics (mean ± standard error) of the bioavailable (Mehlich 1) and semi-total (EPA 3051) concentration of copper and iron in the soil before cultivation of *Canavalia ensiformis*; Supplementary Material S4: Abbreviations.
