# Supplementary material for: From Micronutrients to Potentially Toxic Elements: Physiological Responses of Canavalia ensiformis to Copper and Iron
Source: Metabolites. 2025 Oct 29;15(11):706. doi: 10.3390/metabo15110706 (PMC12654861; doi:10.3390/metabo15110706)
Supplement: Supplementary file 1 [file metabolites-15-00706-s001.zip › metabolites-3878899-supplementary.pdf]

Supplementary Material

Supplementary Material A: Reference concentrations for copper and iron in soil mg Kg^{-1}

A.1. Copper:
In soils, copper values above 25-40 mg kg^{-1}, especially in acidic conditions (pH < 5.5), can induce toxicity, with permissible limits suggested at 20 mg kg^{-1} for soil [16].

A.2. Iron:
Iron typically ranges from 30-550 μg L^{-1} in soil solution, but may exceed 2000 μg L^{-1} in highly acidic soils [16]. Considering a soil bulk density of 1.47 kg dm^{-3}, the iron concentrations of 50 mg kg^{-1} and 141.80 mg kg^{-1} (T50 and T350) correspond to 73,500 μg L^{-1} and 208,446 μg L^{-1}, respectively. These conversions were performed to express iron content in volumetric terms, facilitating comparisons with aqueous iron concentrations established by Kabata-Pendias [16].
The available iron concentration in the soil of the area directly impacted by the iron mining tailings from the Fundão dam ranged from 140.71 to 198.82 mg kg^{-1}, with pH values between 6.72 and 8.32, according to Batista et al. [45]. In partially impacted areas, iron concentrations varied from 134.35 to 263.75 mg kg^{-1}, with pH values ranging from 6.00 to 7.18 [45]. In contrast, the reference area, which was not affected by the tailings, exhibited iron concentrations between 70.86 and 120.37 mg kg^{-1}, with pH values ranging from 3.82 to 5.32 [45]. These variations are attributed to the fact that soil samples were collected in three different locations, located along the Gualaxo do Norte River, in the state of Minas Gerais - Brazil, and also to the fact that there was an increase in soil pH (>5.00) in the areas directly and partially impacted by the deposition of tailings sludge [45].

Supplementary Material B: Statistical packages used in R software:

The packages used in R were: “car” [97]; “cowplot” [98]; “dgof” [99]; “dplyr” [100]; “ExpDes.pt” [101]; “factoextra” [102]; “FactoMineR” [103]; “GGally” [104]; “ggbreak” [105]; “ggfortify” [106]; “ggh4x” [107]; “ggplot2” [108]; “ggpubr” [109]; “ggthemes” [110]; “GOplot” [111]; “multcompView” [112]; “plyr” [113]; “psych” [114]; “qgraph” [115]; “readxl” [116] and “rstatix” [117].

Supplementary Material C

Table C1: Descriptive statistics (mean ± standard error) of the bioavailable (Mehlich 1) and semi-total (EPA 3051) concentration of copper and iron in the soil before cultivation of *Canavalia ensiformis*.

Plant available (Mehlich 1) and semi-total (EPA 3051) concentration of Cu and Fe in soil before cultivation of *Canavalia ensiformis*

TEC	Mehlich1 (mg kg^{-1})	EPA3051 (mg kg^{-1})
Treatment	Cu	
T0	0.58 ± 0.02	1.77 ± 0.32
T50	28.28 ± 1.28	29.08 ± 1.36
T150	87.80 ± 1.67	92.86 ± 4.86
T250	141.97 ± 7.86	145.56 ± 2.67
T350	220.36 ± 2.30	189.43 ± 12.28
Treatment	Fe	
T0	40.91 ± 0.58	5824.07 ± 933.07
T50	56.26 ± 0.53	4653.33 ± 706.27
T150	83.74 ± 1.40	8809.45 ± 1134.16
T250	113.19 ± 0.88	5718.92 ± 1169.29
T350	141.80 ± 2.26	7271.51 ± 689.60

Note: Soil pH of the Cu and Fe treatments was 4.7. No statistical comparisons between means were conducted; p-values and F-statistics are not applicable.

Supplementary Material D: Abbreviations

The following abbreviations are used in this manuscript:

A	Net photosynthesis (μmol CO_2 m^{-2} s^{-1})
ANOVA	Analysis of variance
B	Boron
BNF	Biological nitrogen fixation
Ca	Calcium
Chla	Chlorophyll a
Chlb	Chlorophyll b
CAR	Total carotenoids
CEC	Cation exchange capacity
Ci	Internal carbon (μmol mol^{-1})
CiCa	Ratio between internal and external carbon (mol m^{-2} s^{-1})
ConA	Concanavalin-A
CRD	Completely randomized design
Cu	Copper
CUE	Carbon use efficiency (mol m^{-2} s^{-1})
DMSO	Dimethyl sulfoxide
Dsoil	soil density (kg dm^{-3})
DTPA	Extraction of microelements using chelating solution [49]
E	Transpiration rate (mmol H_2O m^{-2} s^{-1})
EIUA	Intrinsic water use efficiency (μmol CO_2 $mmol^{-1}$ H_2O)
EPA	United States Environmental Protection Agency [52]
EPA3051A	Microwave assisted acid digestion of sediments, sludges, soils, and oils method [52]
Fe	Iron
FEPE	Experimental area of the teaching, research, and extension farm
gs	Stomatal conductance (mmol H_2O m^{-2} s^{-1})

H^++Al^{3+}	Potential acidity
ICP-OES	Inductively coupled plasma optical emission spectroscopy
IUPAC	International Union of Pure and Applied Chemistry [1]
K	Potassium
LA	Leaf area (cm^2)
LAC	Leaves TE accumulation (μg or mg $organ^{-1}$)
LDW	Leaf dry weight (biomass) (g)
LFW	Legume fresh weight (g)
Mn	Manganese
Mg	Magnesium
NFW	Fresh weight of nodules (g)
NL	Number of leaves
NN	Number of nodules
OM	Organic matter
P	Phosphorus
PCA	Principal component analysis
PTEs	Potentially toxic element (s)
RAC	Root TE accumulation (μg or mg $organ^{-1}$)
RAP%	TE accumulation percentage in the root system (%)
RDW	Root dry weight (g)
RFW	Root fresh weight (g)
RL	Root volume (cm^3)
ROS	reactive oxygen species
SAC	TE accumulation in the shoot (leaves + stem) (μg or mg $organ^{-1}$)
SAP%	TE accumulation percentage in the shoot (%)
SB	Sum of bases
SDW	Dry weight of the shoot (leaves + stem) (g)
SFW	Fresh weight of the shoot (leaves + stem) (g)
SL	Stem length (cm)
StAC	TE accumulation in the stem (μg or mg $organ^{-1}$)
StDW	Dry weight of the stem (g)
T0	Control treatment
T50	Treatment 50 mg dm^{-3} of soil
T150	Treatment 150 mg dm^{-3} of soil
T250	Treatment 250 mg dm^{-3} of soil
T350	Treatment 350 mg dm^{-3} of soil
TAC	Accumulation of TE throughout the plant (μg or mg $plant^{-1}$)
TChl	Total chlorophyll
TDW	Total dry weight of the plant (g)
TDWT0	Total dry weight of the plant of control treatment
TE	Trace element
TECleaves	Trace element content in leaves (mg kg^{-1})
TECstem	Trace element content in the stem (mg kg^{-1})
TECroot	Trace element content in the root (mg kg^{-1})
TECsoil	Bioavailable content or semi-total content of TE in the soil (mg kg^{-1})
TI	Tolerance index
TF	Transfer factor
V%	Base saturation
Vsoil	Volume of soil used (dm^3)
WUE	Water use efficiency (mmol CO_2 mol^{-1} H_2O)
